# Another in need enhances prosociality and modulates frontal theta oscillations in young adults

**DOI:** 10.3389/fpsyt.2023.1160209

**Published:** 2023-07-13

**Authors:** Claudio Lavín, Patricia Soto-Icaza, Vladimir López, Pablo Billeke

**Affiliations:** ^1^Centro Interdisciplinario de Neurociencias, Facultad de Medicina, Pontificia Universidad Católica de Chile, Santiago, Chile; ^2^Laboratorio de Neurociencia Social y Neuromodulación, Centro de Investigación en Complejidad Social (neuroCICS), Facultad de Gobierno, Universidad del Desarrollo, Santiago, Chile; ^3^Escuela de Psicología, Pontificia Universidad Católica de Chile, Santiago, Chile

**Keywords:** decision-making, empathy, social cognition, prosociality, electroencephalography, theta rhythm

## Abstract

**Introduction:**

Decision-making is a process that can be strongly affected by social factors. Evidence has shown how people deviate from traditional rational-choice predictions under different levels of social interactions. The emergence of prosocial decision-making, defined as any action that is addressed to benefit another individual even at the expense of personal benefits, has been reported as an example of such social influence. Furthermore, brain evidence has shown the involvement of structures such as the prefrontal cortex, anterior insula, and midcingulate cortex during decision settings in which a decision maker interacts with others under physical pain or distress or while being observed by others.

**Methods:**

Using a slightly modified version of the dictator game and EEG recordings, we tested the hypothesis that the inclusion of another person into the decision setting increases prosocial decisions in young adults and that this increase is higher when the other person is associated with others in need. At the brain level, we hypothesized that the increase in prosocial decisions correlates with frontal theta activity.

**Results and Discussion:**

The results showed that including another person in the decision, setting increased prosocial behavior only when this presence was associated with someone in need. This effect was associated with an increase in frontocentral theta-oscillatory activity. These results suggest that the presence of someone in need enhances empathy concerns and norm compliance, raising the participants’ prosocial decision-making.

## Introduction

In everyday life, there are several examples of decisions that could have direct consequences on others, such as deciding how much money to donate to charity, how much food to buy when there are supply problems, or whether to give back a wallet full of cash that has just fallen from someone’s pocket. In all these cases, people may consider, to different extents, the consequences of their behavior on the wellbeing of others and themselves (1). This processing is crucially affected by the circumstances in which the decision has to be made (2). For example, to be observed by others or to observe someone in physical pain or distress changes our tendency to consider others’ well-being in our decisions (3, 4). Although the influence of others seems clear, the psychological and neurobiological mechanisms underlying this process remain unclear.

Prosocial behavior refers to any action that is addressed to benefit another individual (5), and in its extreme form of altruism, it implies a costly act that confers benefits to others (6). Although traditional economics approaches assume a primarily selfish impulse of the maximization of immediate utilities in every human decision (7), evidence has shown a much more complex scenario in which social and emotional factors, together with concerns about their own and others’ wellbeing, explain people’s social preferences (5, 6). Indeed, consistent evidence has shown that prosocial behavior can be associated not only with direct and indirect benefits to the decision-maker but also with his/her concerns about others’ wellbeing. In this line, empathy, defined as the sharing and comprehension of others’ feelings and thoughts (8), is an important modulator of prosociality by enhancing the presence of the other within the decision setting (8).

There is evidence showing the impact that the presence of others has on subjects’ decisions. The way other persons appear varies depending on the experimental settings and goes from eyespots on the wall observing people’s decisions to helping others under “real” physical pain. Evidence comparing public and anonymous decisions has described that individuals behave more prosocially and adjust their behavior to a norm while being observed (9–18). This evidence has suggested that, through emotional resonance and perspective-taking, the representation of another person can influence both decisions and brain activity associated with that process. Accordingly, different experimental manipulations have modulated the presence of another person in the decision-making setting through variations in both the level of empathy-related responses and the reputational value of the decisions.

The relationship between empathy and prosociality has been well-established by behavioral and brain studies. The evidence relates prosocial behavior and emotional resonance in children (9, 19, 20), also relates cooperation rates and empathetic perspective-taking in women (21, 22), and empathy states (associated with seeing others in need or pain) has been related to prosocial behavior (23). Moreover, the perception-action model proposed empathy as an important factor in explaining altruistic behavior (24). Neuroimaging evidence shows that in experimental tasks involving observing others in pain, brain activity overlaps with areas usually required during decision-making tasks, depending on task context. These structures include the anterior insula (AI), and the midcingulate cortex [MCC, commonly referred to as the dorsal anterior cingulate cortex, see (25)]. Specifically, AI activity has been related to being more prosocial toward people perceived as victims of pain (10), AI and MCC activity has been related to helping behavior (12). There is also electroencephalographic (EEG) evidence showing oscillatory activity related to the presence of others with different degrees of empathy involvement. For instance, increased frontal theta activity has been related to observing others in pain (compared to neutral stimuli) as well as to one’s own unpleasant feelings (26, 27). Frontal theta activity is also related to self-reflexive thinking within social interactions (28) and predicting others’ decisions (20, 29). These data support the relevance of the presence of others in people’s decision-making and that there are different ways in which others are included in the variables that a decision-maker takes into account while deciding.

Prosocial decisions can also indirectly benefit decision-makers in the form of reputation. There is evidence showing the tendency of people to cooperate (or defeat) with those who cooperated (or defeat) with them in the past (known as direct reciprocity) (14) and to help those who help others (known as indirect reciprocity) (30, 31). This latter form of reciprocity is proposed as the reason why it would pay to develop a prosocial reputation. Thus, the presence of an observer might influence prosociality related to the potential benefits that this behavior provides in the form of reputation and eventual reciprocity. For instance, when dictators make their offers facing eyespots on the screen as an indicator of observability, they increase their offers over the expected (32). There is evidence also showing an increase of the prosociality rates in the dictatorial game (DG) when participants know the name of the person they are interacting with, which is a form of suppressing the anonymity of the other player (33). Moreover, participants who perform the DG and the ultimatum game (UG) propose significantly fairer offers in the UG than in the DG condition (34). These fairer offers within the UG setting were accompanied by higher activation in the right dorsolateral prefrontal cortex (dlPFC) and the ventromedial prefrontal cortex (vmPFC). The involvement of these areas suggests predictions about possible rejection by recipients (35, 36) and the inhibition of immediate self-oriented responses to obtain higher final payoffs (37–39). Brain models of social cognition have shown different networks related to activity in the temporoparietal junction (TPJ) and in the dorsomedial prefrontal cortex (dmPFC) related to inferring another’s mental states, and the amygdala and vmPFC related to the affective value of social behavior (40–48). Recent evidence has shown the relevance of the MCC in social cognition, particularly given its connectivity to the listed regions and its modulation to other-oriented information (40, 49).

Based upon the previously mentioned evidence, the present study aims to assess, through a slightly modified version of the DG, the impact that the presence of another person has on the prosocial decisions of participants. We studied the behavior and brain activity of adult individuals while playing a modified version of the DG within three different scenarios: when the decisions were anonymous (control condition, CC), when participants were observed by another person (observer condition, OC), and when the decisions were in the context of another person who is in need (empathy condition, EC). These contextual manipulations aimed to modulate the intensity of the empathy concerns triggered by the presence of another person within the decision setting. We tested two hypotheses: (i) the presence of another person increases prosocial decisions, and this increase is higher when this person is in need (EC), and (ii) the increase in prosocial decisions is correlated with frontal theta activity.

## Materials and methods

### Participants

Thirty-two healthy participants (13 women) with normal or corrected-to-normal vision, without a history of neurological or psychiatric diagnosis, performed the experimental task. Their age range was between 19 and 23 years old (*M* = 21.4, SD = 1.3). Participants were randomly assigned to one of the two experimental conditions (see experimental task below). The experimental procedures were performed in accordance with institutional guidelines and were approved by the Ethical Committee of the Pontifical Catholic University of Chile. All participants gave their written informed consent. All experiments were carried out at the Social Neuroscience and Neuromodulation Laboratory of the Social Complexity Research Center (neuroCICS), Universidad de Desarrollo, Santiago, Chile.

### Power and sample size

To calculate the minimum sample size and the power of the current study, we used the behavioral effect as the primary outcome. A similar study related to the effect of the presence of others during socioeconomic decision-making (32) shows an effect size of *η*^2^ = 0.149, which is a large effect (50). Taking into account publication bias, we set an intermediated effect size of *η*^2^ = 0.1. Thus, for the between-within factor interaction in a 2×2 mixed ANOVA with a power of (1-β) = 0.95 and a significance level of *α* = 0.05, the minimum sample size to find the expected effect was *n* = 32.

### Experimental task

The experimental task consisted of a modified version of the DG, in which participants had to choose between two possible allocations of money for themselves and for another player (hereinafter the other). Participants were told that the other player was a future participant of the experiment. Within the two possible choices, there was always one that gave participants the option of being prosocial. In the case of this task, a prosocial decision is defined as choosing the option that provides higher income to the other player regardless of the consequences for the participant. At the end of the experimental task, one trial was randomly selected and the choice made by the participant in that trial determined both the amount of money that he/she received, and the amount left to the future participant (the other). All participants were informed that they would receive an amount of money at the end of the experiment, which would correspond to the sum of two factors. One is composed of one of their own decisions (this is, the amount chosen in the randomly-selected trial), and the other corresponds to a decision made by a previous player, also selected at random. In the latter case, the amount corresponds to the money that a previous participant paid to his/her other player during his/her performance of the task. This design has the aim of highlighting that decisions have real consequences for both the participants themselves and another person.

The sample was randomly assigned to one of the two groups: the empathy group and the observer group. For both groups, participants first performed a round of the task as a control condition, in which their decisions were told to be anonymous. After this first round, participants assigned to the empathy group performed a second round of the task under EC. We maintain this fixed order to prevent any susceptibility effects that may bias the decision in a control condition if an experiential manipulation occurs before it. The EC is the same task as in the CC, but at this time, participants were told that this part of the experiment was part of a larger study that involved donations of money to real charity institutions, but that these donations did not depend on the specific decision of the participants. They were informed that, under this condition, the decisions were anonymous (as in the CC). It is important to note that, at this point, all the participants received explicit clarification that the amount of money that they allocated was not a direct donation to the charity institution. No further information was given about the charity institution, and a blurred picture of a child who would benefit from this donation was shown to the participants during the rest of the experimental task. Regarding the observed group, after playing the CC, participants played a round of the task under the OC. The OC is the same task as in the CC, but at this time, participants’ were told that their decisions were observed remotely by another researcher, who was part of the research team, so their decisions were no longer anonymous. They were informed that the observer was a sociologist who conducted the observation as part of another study. A blurred picture of the observer was shown to the participants for the rest of the experimental task (as in the EC). Moreover, a confederate playing the role of a sociologist followed a predetermined script. At the beginning of the OC, the confederate introduced herself to the participants. It is important to note that the confederate was not physically present during the actual participant decisions. The two conditions (i.e., EC and OC) were designed to observe two variables that have been reportedly influential over prosocial decisions: empathy concerns and social norm-compliance. By making the decisions anonymous during the EC, we could isolate the empathy concerns related to the charity institution (and the child potentially benefited by the donation), and by making the OC non-anonymous we could isolate the social influence of making decisions under observation.

**Figure 1:**
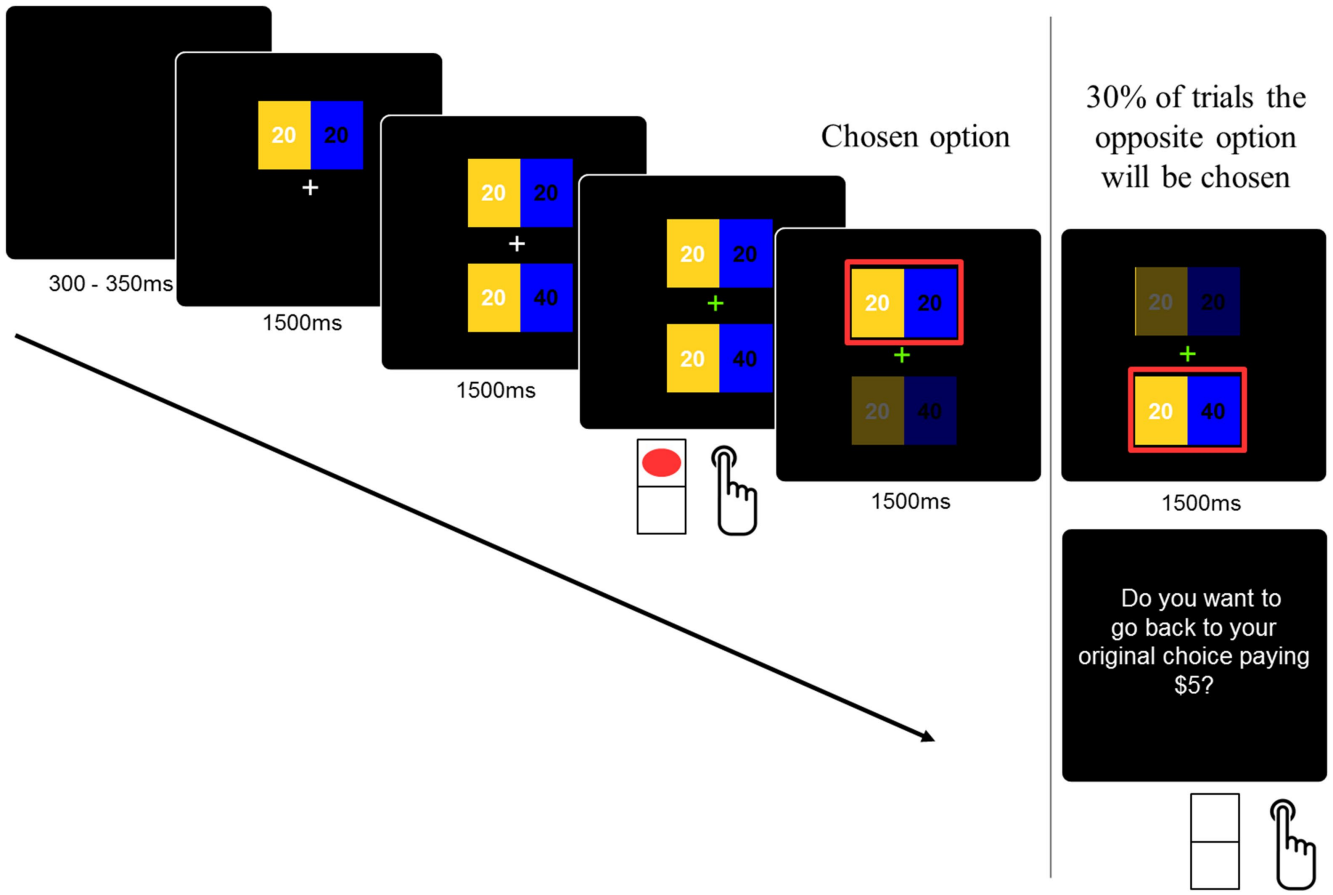
The experimental task. The decision task consisted of selecting one within two distributions of money that were presented in the higher and lower parts of the screen. Both allocations involved money for the participant and for another player. The other player was identified as a future participant in the experiment. Participants had to press the bottom up or down according to their choice on a normal computer keyboard. The order of the options was randomized.

The decision task consisted of selecting one within two distributions of money that were presented in the higher and lower parts of the screen. These distributions or allocations were presented separately for 1,500 ms each, as shown in Figure 1. The reason for separating the options is to ensure time-locked processing of the social dilemma. Both allocations involved money for the participant and another player. The other player was identified as a future participant in the experiment. The amounts for participants were presented on the left side of the screen in yellow, and the amounts for the other player were presented on the right side of the screen in blue (see Figure 1). The amounts were presented for 1,500 ms, and a visual cue (green fixation cross) indicated to the participants that the decision could be delivered. The experimental design considered the counterbalance of the position in which the stimulus was displayed (up/down) on the screen. Moreover, it was randomized whether the prosocial option appeared first. The color assignment (participant/other) was not counterbalanced in order to avoid confusion in decision-making during the task.

**Figure 2:**
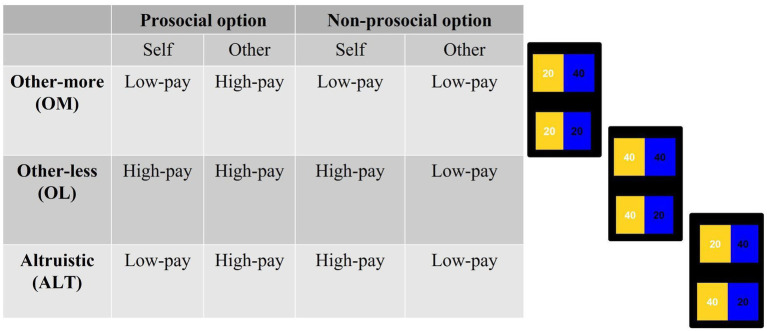
Structure of distributions presented to participants during the experimental task. The order of the options was randomized.

During all conditions, participants faced three types of cases that were randomly presented: other-less (OL), other-more (OM), and altruistic (ALT) (see Figure 2). Both in the OL and in the OM cases, the prosocial option has no personal costs to the players. Specifically, in the OL case, the prosocial option prevents the other participant from obtaining lower earnings, whereas in the OM case, the prosocial option involves higher earnings for the other participant. On the contrary, in the ALT case, the prosocial option involves a personal cost to the participant.

Finally, participants were told that sometimes the game would choose the opposite option to the one chosen by them (e.g., if the participant chose the prosocial option, then the non-prosocial option would be displayed as the chosen option). If they want to return to their original option, they will be punished in that trial by losing a fixed amount of USD$5, but if they want to keep the “error,” then that choice (the opposite of the one they wanted) will be considered. This was used as a way to confirm the strength of participants’ decisions. At the end of the experimental task, all participants received a payment from one of the played trials (selected at random) plus what a previous participant left to them.

### EEG

Brain activity was recorded from 64 scalp electrodes using a Brain Vision amplifier system (BrainProducts, Germany, electrode impedance <5 kΩ, 0.15–500 Hz, 1,000 samples/s). EEG data were segmented into epochs centered around the presentation of the second offer (from −0.5 to 1.4 s) when the dilemmas were presented, and the two options became clear. All recorded EEG epochs were individually checked for artifacts by visual inspection. Artifacts were first automatically detected using a threshold of 150 μV or 3 Std. Dev. and a power spectrum greater than 2 Std. Dev. for more than 10% of the frequency spectrum (1–30 Hz). Blinking was extracted from the signal by means of ICA. Trials that included artifacts detected automatically and confirmed by visual signal inspections were eliminated. The artifact-free EEG material was recomputed to average reference and digitally bandpass filtered to 0.1–45 Hz. Whole power distribution was computed using Wavelet transform, with a 5-cycle Morlet wavelet, in a −1.5–1.5 s window around the onset of the second offer. For all analyses, we used the dB of power related to the baseline (15 s acquired at the beginning of each block).

### Statistical analysis

We used the Kolmogorov-Smirnoff test for normality. When the data did not meet the normal assumption, we used nonparametric tests. For the EEG statistical analysis, we first fitted a general linear model (GLM) of the power of the oscillatory activity per trial in each participant [first-level analysis, see (51–55)] using the following equation:

$\begin{array}{l} \text{Power}\left(t,f\right)=\beta_{1}+\beta_{2}\Delta A1+\beta_{3}\;\Delta \mathrm{A2}+\beta_{4}\;\Delta A1^{*}\Delta \mathrm{A2}+\beta_{5}T\\ \;\;\;\;\;\;\;\;\;\;\;\;\;\;\;\;\;\;\;+\beta_{6}\Delta A1^{*}T+\beta_{7}\;\Delta A2^{*}T+\beta_{8}\;\Delta A1^{*}\Delta A2^{*}T\left(1\right) \end{array}$

where β_1_ is the intercept, β_2_ is the slope (coefficient) of the variable ΔA1 (differences between the allocation for the player and the other in the first presented distribution), β_3_ is the slope of the difference between the allocations for the player and the other in the second presented distribution (ΔA2), and β_4_ is the slope of the differences between the allocation for the player and the other in both offers (note that this regressor takes values other than zero only in the ALT case). Additionally, we added a regressor for the experimental condition (T, which takes the value 1 when the decision is made during the experimental manipulation, observer, or empathy conditions, and 0 in the control condition) with its respective slope (β_5_), together with the interactions between the experimental condition and the other regressors (ΔA1*T, ΔA2*T, and ΔA1*ΔA2*T). Then, we obtained a 3D matrix of the normalized β-values estimated (electrode, time, frequency, β-value/standard error) for each regressor and participant. We then explored for differences between groups (observer and empathy) and conditions (control and experimental) using the Wilcoxon test (second-level analysis).

To investigate modulations within and between groups, we initially focused on a preselected group of electrodes of interest. Based on prior research (56), this group consisted of the following frontal electrodes: Cz, FCz, C1, C2, FC1, and FC2. For this analysis, we computed the average of the 3D matrix of normalized β-values across the electrode dimension, using only the selected electrodes, before conducting the statistical comparison. Additionally, we performed a whole-scalp analysis using the 3D matrix of each subject to explore modulations outside the frontal regions. We applied the cluster-based permutation (CBP) test (57) for correcting multiple comparisons in both the frontal electrodes and whole-scalp analyses. Briefly, in this method, the clusters of significant areas were defined by pooling neighboring sites (in the time-frequency chart and adjacent electrodes) that showed the same statistical effect (cluster threshold detection, CTD, uncorrected *p* < 0.05). The cluster-level statistics were computed as the sum of the statistics of all sites within the corresponding cluster (e.g., Z value for Wilcoxon test). We evaluated the cluster-level significance under the permutation distribution of the cluster with the largest cluster-level statistics. The permutation distribution was obtained by randomly permuting the original data (i.e., permuting a specific regressor per trial for within-subject analyses or group labels for between-subject analyses). After each permutation, the original statistics test was computed (i.e., the Wilcoxon test), and the cluster-level statistics of the largest resulting cluster were used for the permutation distribution. After 5,000 permutations, the cluster-level significance for each observed cluster was estimated as the proportion of elements of the permutation distribution larger than the cluster-level statistics of the corresponding cluster.

To investigate the association between brain activity and prosocial behavior, we conducted a mediation analysis employing the MediationToolbox for Matlab. This analytical approach enabled us to explore the mediating role of brain activity in connecting our dependent variable, prosocial responses, with the independent variables related to the experimental manipulations. We employed bootstrapping to calculate statistical significance.

### Source analysis

We used Brainstorm software to estimate neural current density time series at each brain location using a minimum norm estimate inverse solution LORETA algorithm with unconstrained dipole orientations in single-trial signal per condition and subject. We used a five-layer continuous Galerkin finite element conductivity model and a physical forward model. The estimated source current at the cortical surface was obtained by multiplying the recorded raw EEG time series at the electrodes by the inverse operator. Time-frequency analysis was then conducted on the source space directly using Morlet wavelet transform. Only statistically significant differences at electrode and source levels, surviving multiple comparison corrections, were presented.

## Results

### Behavioral results

To assess participants’ decisions, a mixed ANOVA was conducted using Case (type of offers, i.e., ALT, OM, or OL) and Condition (control or empathy/observer) as independent variables and prosocial responses as the dependent variable. By prosocial response, we mean the proportion in which subjects choose the option that benefits (or does not punish) the other player. The variable Group (i.e., observer or empathy) was used as a between-subjects factor.

Significant main effects of case and condition (see Table 1) and the interaction between condition and case were found. This suggests that participants tended to cooperate differently depending on the case. A between-subjects interaction with Condition, Case, and Group was also found.

**Table 1:** Mixed ANOVA with case, condition, and group as independent variables and response as the dependent variable.

	Sum of squares	df	Mean squares	*F*	*p*	*η* ^2^
Condition Residual	0.900 1.966	1 39	0.900 0.050	17.857	<0.001	0.31
Case Residual	23.473 10.414	2 78	11.736 0.134	87.905	<0.001	0.693
Condition * Case Residual	0.224 2.216	2 78	0.112 0.028	3.950	0.023	0.092
Condition * Case * Group (between subjects) Residual	0.302 1.913	2 76	0.151 0.027	6.004	0.004	0.06

**Figure 3:**
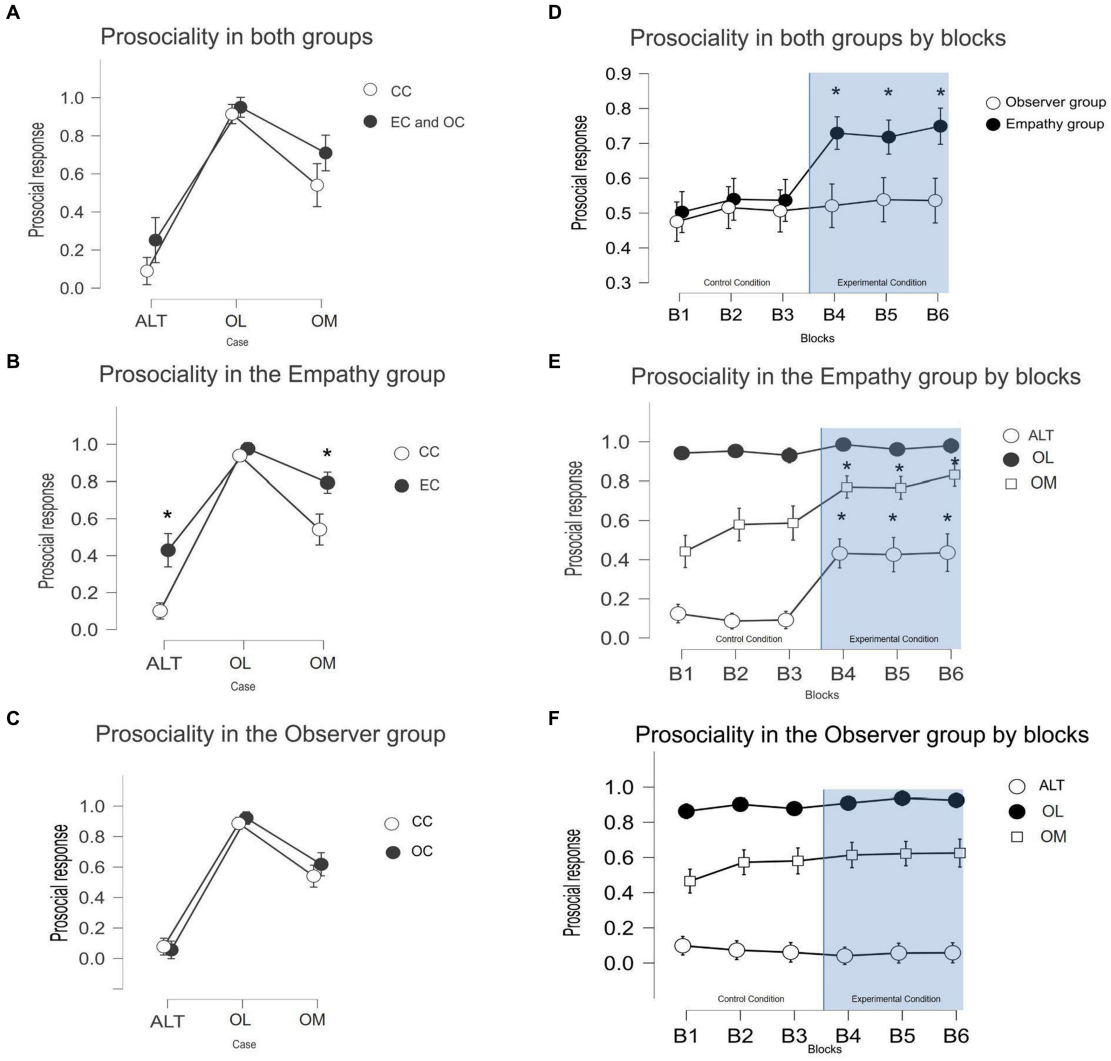
**(A)** Means and standard deviation of the cooperation rates across each 3 cases by both groups collapsed. **(B)** Means and standard deviation of the cooperation rates across the 3 cases in the empathy group. **(C)** Means and standard deviation of the cooperation rates across the 3 cases in the observer group. **(D)** Means and standard deviation of the prosocial choices across 6 blocks of trials during the experiment. Block 4 is the first block of the experimental condition across both interventions. **(E)** Means and standard deviation of the cooperation rates by case across 6 blocks of trials in the Empathy group. **(F)** Means and standard deviation of the prosociality rates by cases across 6 blocks of trials in the observer group.

Participants in both groups tended to be less prosocial in ALT cases than in the other two cases (*post hoc* ALT-OM: p(bonf) < 0.001, *t* = −7.891, Cohen’s *d* = −2.087, *p*-values adjusted for comparing a family of 15). This means that when the prosocial options involved personal costs, participants decided to choose it less than when there were no costs associated with this choice. Interestingly, participants decided to be less prosocial in OM cases than in OL cases (*post hoc* OL-OM: p(bonf) < 0.001, *t* = 3.230, Cohen’s *d* = 0.839), even though in both cases, there were no personal costs involved in their decisions. This means that participants decide to be more prosocial when this prevents other individuals from earning less than them but were less prosocial when the other participants could earn more than them in the trial (see Figure 3A).

The separate analysis of both groups shows an effect of the EC but not of the OC (see Figure 3). Within the observer group (Figure 3C), there was no effect of the experimental intervention on the prosociality rates [*post hoc* control (c) ALT - experimental (e) ALT: p(bonf) = 1, *t* = 0.384; *post hoc* cOL-eOL: p(bonf) = 1, *t* = −0.622; *post hoc* cOM-eOM: p(bonf) = 0.9, *t* = −1.38, *p*-values adjusted for comparing a family of 66]. Within the Empathy group (Figure 3B), there was an effect of the experimental intervention in both ALT and OM cases where the prosociality rates increased relative to the control condition (*post hoc* cALT-eALT: p(bonf) < 0.001, *t* = −4.153, cOM-eOM: p(bonf) < 0.001, *t* = −6.153). There were no differences between the OL cases (cOL-eOL: p(bonf) = 1, *t* = −0.695). The prosociality rates in the OL case were high in the control condition across both experimental interventions. The between-subject effects show no differences in the cooperation rates between the two control conditions in all cases (p(bonf) = 1 for the three cases).

We conducted a temporal analysis of the prosocial choices to investigate potential bias related to the order of the experimental condition (see Figure 3D). To observe the changes in prosocial choices across the experimental task in more detail, the control and experimental conditions were divided into three blocks of trials each. The variable blocks and cases were used as independent variables, and prosocial responses were used as dependent variables. Within the Empathy group (Figure 3E), the results confirm the effect of the experimental intervention [*post hoc* block3-block4: p(bonf) < 0.001, *t* = −6.008] but not in the Observer group (Figure 3F) [p(bonf) = 1]. Prosocial choices within the empathy group remained stable across the last 3 blocks of the EC [*post hoc* block4-block5: p(bonf) = 1; block5-block6: p(bonf) = 1].

As mentioned previously, during the experiment, 30% of trials indicated that the game chose the opposite option as the one chosen by participants, who had the option to return to their original choice at a monetary cost. We analyzed whether there were differences between the control and experimental conditions in the proportion in which participants maintained or changed their options given their initial choice. The dependent variable for the analysis was the proportion in which subjects maintained a prosocial choice (this means, if they chose the prosocial choice, the game inverted their decisions, and then participants returned to their original prosocial election) and the proportion in which participants changed their original non-prosocial choice to the prosocial choice selected by the game.

**Figure 4:**
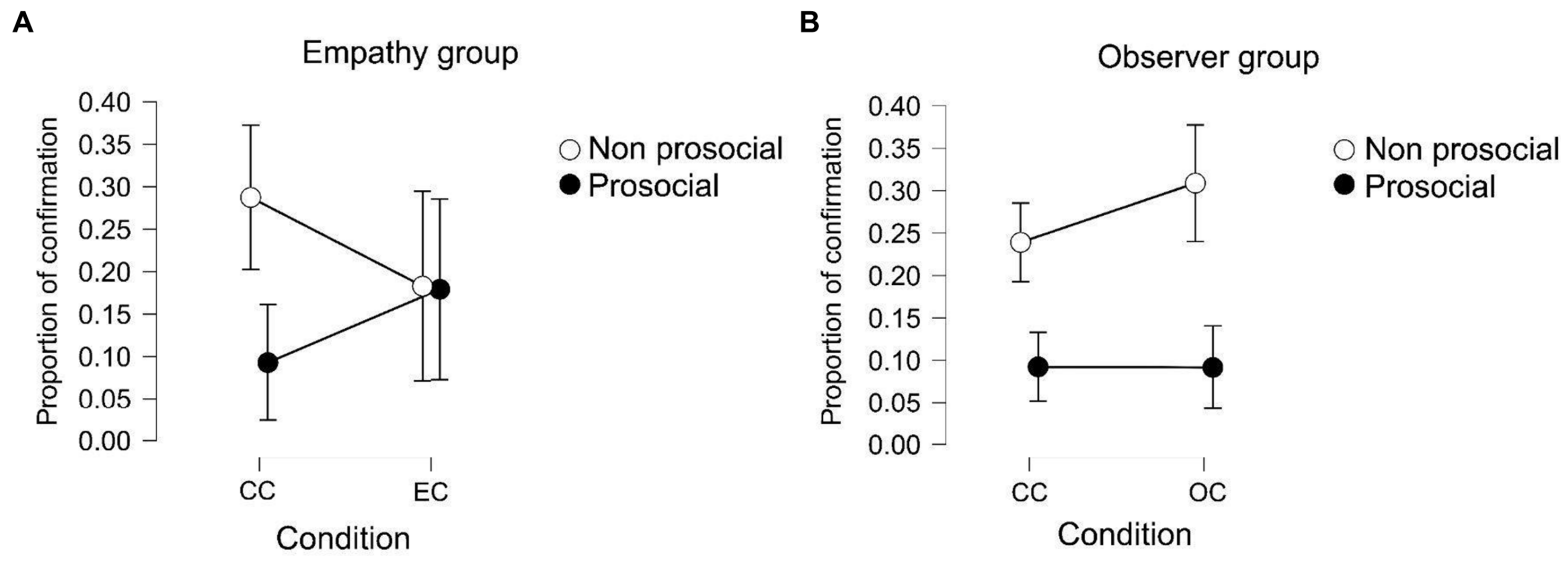
**(A)** Proportion in which participants of the empathy group confirm their original choice. The white dots represent the choice that participants took in the first place, in white non-prosocial and in black prosocial. **(B)** Proportion in which participants of the observer group confirm their original choice.

A repeated-measures ANOVA was conducted using group, experimental conditions, and offers as independent variables and the proportion of confirmatory prosocial choices as the dependent variable. The results (see Figure 4) show an effect of prosociality (*p* < 0.001, *F* = 19.572, *η*^2^ = 0.177) on the interaction between group and prosociality (*p* = 0.046, *F* = 4.263, *η*^2^ = 0.038) and on the interaction between condition, group, and prosociality (*p* = 0.003, *F* = 10.098, *η*^2^ = 0.028).

*Post hoc* analyses show that participants within the observer group tend to confirm their non-prosocial choice more than their prosocial choice in both control and experimental conditions [*post hoc* pro-nonpro p(bonf) < 0.001, *t* = 4.527]. This means that when participants chose the non-prosocial option in the first place, but the opposite was presented (as if they had chosen the prosocial option), they confirmed their non-prosocial choice in a higher proportion than when they chose the prosocial option in the first place. In the empathy group, there were no differences between the control and experimental conditions. The between-subject effect shows that there were no differences between the groups in either condition.

### Brain results

A time-frequency analysis was conducted to observe the effect of the differences in the spectral activity, particularly in the theta-band oscillatory activity, relative to the unequal monetary distributions during the OL and OM cases. As mentioned above, consistent evidence shows that theta oscillatory activity is related to self-inhibitory activity and the encoding of social cues during decision processes. This analysis was conducted given the behavioral differences between the OL and OM cases within the empathy group. These differences show that while participants decided to be similarly prosocial in the OL cases in both control and experimental conditions, there were higher rates of prosociality in the OM experimental condition. This is interesting given that, in both cases, the players choose between options with the same earnings for themselves, but while in the OL case, the prosocial choice prevents the other player from receiving less than them, in the OM case, the prosocial choice involves that the other player receives more than them. Thus, we first fitted a GLM of the power of the oscillatory activity per trial during the presentation of the second offer. As a regressor, we used the differences in the earnings between the participants themselves and the other player for the first and the second offers separately and the interaction between them. Note that the interaction captures the variance of the signal given by ALT cases (see method); thus, the other regressors capture the variance given by the other cases (OL and OM). Specifically, these regressors capture the difference between OL and OM when no equal offers are presented in the first distribution (ΔA1) or the second distribution (ΔA2). We mainly focused on exploring the different modulations in these regressors for experimental conditions (using the interaction: ΔA1*T and ΔA2*T, see methods). For the second-level analysis, we pooled both regressors (ΔA1*T and ΔA2*T) to capture the variance related to the decision processing when facing these cases under experimental conditions. Thus, we explored differences between groups and conditions using the Wilcoxon and CBP tests (see Methods for more details).

**Figure 5:**
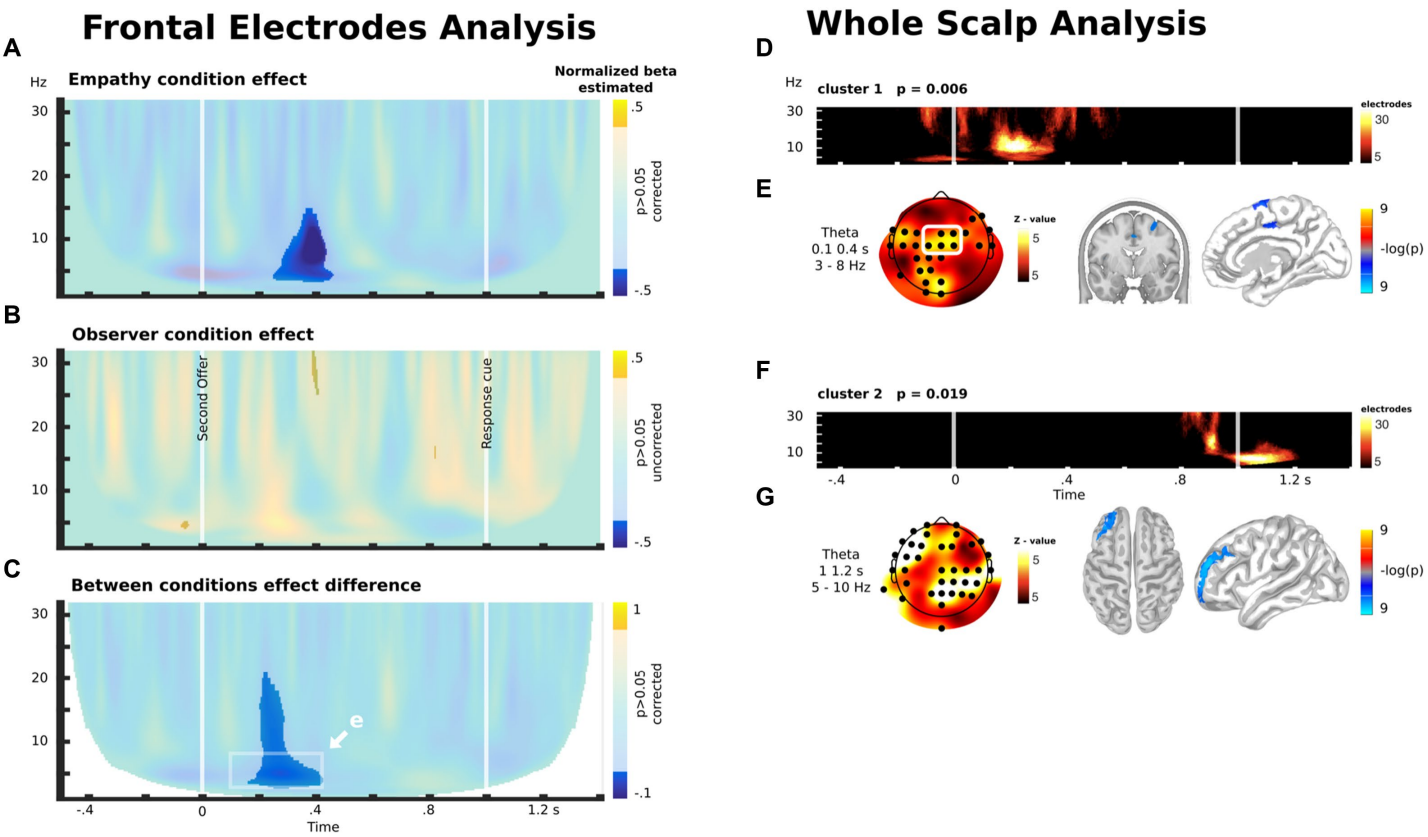
**(A)** Difference between the control and empathy conditions in the correlation between oscillatory brain activity in the frontocentral electrode (white rectangle in **E**) and the unequal distribution to the other player during the decision time. **(B)** Difference between the control and observer conditions in the correlation between the oscillatory brain activity in the frontocentral electrode (white rectangle in **E**) and the unequal distribution to the other player during the decision time. **(C)** Between-group comparison of the difference between the control and experimental conditions in the correlation between the oscillatory brain activity in the frontocentral electrode (white rectangle in **E**) and the unequal distribution to the other player during the decision time. **(D)** Significant cluster emerging by means of exploratory whole-scalp analysis for the contrast shown in **C**. **(E)** Scalp distribution of the theta modulation in the time-frequency windows highlighted in C and its source estimation. **(F)** Another significant cluster emerging by means of exploratory whole-scalp analysis for the contrast shown in **C**. **(G)** Scalp distribution of the theta modulation of the cluster shown in **F** and its source estimation. **(D,F)** The color scale represents the number of electrodes with significant activity within each time-frequency bin, as part of the cluster.

Based on prior research (56), we first explored modulation in frontal electrodes (depicted in the white rectangle in Figure 5E, see Methods). For the empathy group, we found a negative modulation of theta/alpha activity approximately 300 ms after the second offer (Figure 5A). In other words, there was an increase in theta/alpha activity when participants faced OM cases (in comparison with OL cases) in the experimental (empathy) condition. For the observer group, we did not find any significant modulation (Figure 5B). These results led to a significant difference in frontocentral theta oscillatory activity modulation between the Empathy and Observer groups during the experimental intervention (Figures 5C,E). These results indicate that EC, but not OC, led to an increase in frontocentral theta oscillatory activity when participants were exposed to cases in which the other player could earn more than themselves.

These results were corroborated using a whole scalp analysis without *a priori* assumptions about the localization of the brain modulation between groups (Figure 5D). This analysis shows the emergence of a consistent cluster that overlaps with the activity found in the preceding analysis. Interestingly, the analysis also showed another modulation in theta/alpha oscillatory activity when participants watched the cue that indicated that the decision had to be delivered (Figures 5F,G). Source analysis indicated that theta modulation after the second offer are located in the MCC and right superior frontal gyrus (r-SFG) (Figure 5E), while the modulations after the response cue are located in the left middle frontal gyrus (l-MFG, or dlPFC) (Figure 5G).

Finally, we explored whether theta/alpha activity after both the second offer and the response cue were related to the rate of prosocial choices. For this, we correlated the normalized b-value per subject in both the r-SFG after the second offer, and in the l-MFG after the response cue with the rate of prosocial decisions in OM cases during the experimental conditions. The Spearman partial correlation showed that prosocial decisions in OM cases during the experimental condition correlated with theta activity in the r-SFG pooling both groups (*r* = −0.37, *p* = 0.03, *n* = 32) and not with theta activity in the l-MFG (*r* = 0.1, *p* = 0.5, *n* = 32). When analyzing the groups separately, we found a significant correlation in the observed group (r-SFG: *r* = −0.57, *p* = 0.02, *n* = 16; l-MFG: *r* = 0.3, *p* = 0.2, *n* = 16), but not in the empathy group (abs(r) < 0.04, *p* > 0.8, *n* = 16). To explore this with more detail, we carried out a robust linear regression for the rate of prosocial decisions in the OM cases for experimental conditions. In accordance with the preceding result, the relation between theta activity in the r-SFG and prosocial decisions differed between the observer and empathy groups (see Table 2).

**Table 2:** Robust linear regression of the prosocial decision in the OM case during experimental conditions for both groups.

	Beta	S.E	*t*-value	*p*-value
Int	0.39	0.08	4.4	0.0001
Theta (l-MFG)	0.03	0.03	1.05	0.3
Theta (r-SFG)	−0.13	0.05	−2.3	0.027
Observer condition (OC)	0.14	0.1	1.4	0.17
Theta (r-SFG)*OC	0.21	0.09	2.2	0.035
Theta (l-MFG)*OC	−0.02	0.07	−0.36	0.7
OM control condition	0.64	0.12	5.2	<0.0001

**Figure 6:**
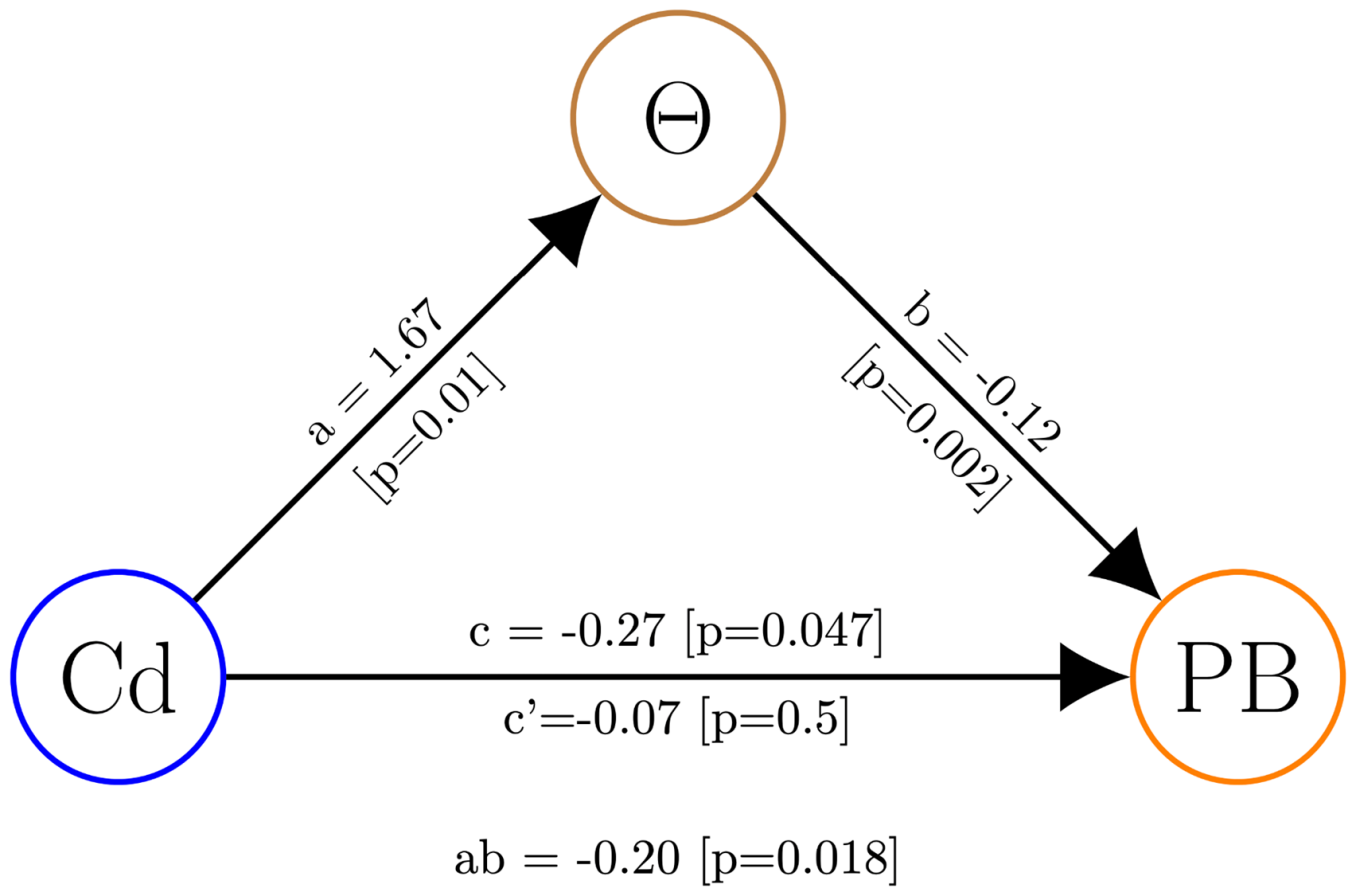
Mediation analysis with theta activity in the r-SFG (Θ) as the mediating variable. The experimental conditions (Cd) served as the independent variable, while prosocial behavior in OM cases (PB) was the dependent variable.

Finally, to assess whether theta activity plays a mediating role in the influence of the experimental intervention on prosocial behavior, we conducted a mediation analysis using theta activity in the r-SFG as a mediating variable. The results of our analysis revealed a significant mediation effect, indicating that theta activity serves as a mediator in this relationship (see Figure 6).

Taken together, these results show that the empathy intervention increases prosocial decisions modulating frontal theta activity when evaluating the different options and when making the decision. The observer intervention did not modulate the prosocial decision on the whole group of subjects, but those subjects that increased their prosocial decision demonstrated a theta modulation that was similar to the one that the subjects in the empathy group showed.

## Discussion

Based on previous experimental results, we hypothesized that the presence of another person would increase prosocial decisions and that this increase would be higher when the other person is associated with others in need (EC). At the brain level, we hypothesized that the increase in prosocial decisions correlates with frontal theta activity. The results showed a differential effect between the two experimental conditions (OC and EC) in behavior and brain activity. While the empathy group showed increased prosocial decisions during the experimental condition associated with a modulation of frontal theta activity, there were no effects in the observer group. The brain activity and behavioral change found in the EC can be interpreted as an enhancement of the saliency of someone in need and/or an increase in social norm compliance.

Brain results show that the inclusion of someone in need had an effect on the way in which participants faced the decision with favorable inequity to the other player (OM cases). Oscillatory activity shows that this experimental intervention increased frontal theta activity relative to the unequal favorable distribution to the other player. The source of this activity is compatible with the posterior part of the MCC and superior frontal gyrus. The MCC has been traditionally associated with several processes that usually are recruited during decision-making according to the specific cognitive demands and contextual factors, particularly error detection, self-monitoring, socially driven interactions, and empathy-related responses (51, 53, 58–60). There are reports of a general role of the MCC in the processing of multimodal context-dependent events (61, 62), which has been specifically observed in pain-related experiments that modulate empathy responses (63–68). Macaque studies, moreover, have shown that lesions to the anterior cingulate gyrus decreased the value that those animals give to social stimuli (40) and that activity in this area is related to the observation of rewarding outcomes delivered to another animal (49). Human studies have shown that the MCC processes cues that inform the motivational state of another person, providing the interpretation that this area participates in monitoring others’ behavior for learning about and from others (40). Taking our results in context to the presented evidence, we can suggest that the frontal theta activity introduced by the empathy condition reflects the participants’ detection of relevant social information from the other person in need. Although the person in need in the EC does not benefit directly from the participants’ decisions, recruiting the preceding brain mechanism can increase the participants’ prosocial decisions. There is evidence about incidental emotions, which are emotions unrelated to an ongoing task, as a factor that can influence decisions (69). We can interpret that the presence of someone in need enhanced an empathy state in participants that favored the election of the prosocial choice. It is important to note that this effect was not present during the OC, although another person was also in the decision setting. Interestingly, participants who increased their prosocial choices in the OC showed a theta modulation similar to the EC. The stronger effect observed during EC can be due to emotion-related processing elicited by the inclusion of the other in need into the decision setting. Indeed, the posterior part of the MCC has been extensively associated with empathy/emotional resonance (25, 63, 70).

Another possible interpretation of the findings is that including another person in need modulated a more general compliance-to-the-norm response. Subjects across both groups tended to be less prosocial when personal costs were associated with the prosocial choice. This is in line with previous evidence showing lower rates of prosocial decisions in the dictator game (DG) than in the ultimatum game (UG) (71–73). While in the former game, there are no monetary incentives for prosociality, in the latter game, subjects need to consider others’ earnings to obtain higher outcomes. Our results show that participants tended to be more prosocial when this prevented other participants from earning less than them but were less prosocial when this led the other to higher earnings. Our brain results show an increase in frontal theta activity relative to the unequal favorable distribution to the other player. This activity has been previously associated with fair behavior in the DG and has been interpreted as cognitive control and self-inhibition (56, 60). Evidence shows the medial-prefrontal activity related to the rejection of unfair offers in the UG (74) and specific insular activity related to unfair rejections to third parties. This frontal activity associated with cognitive control and inhibition is in line with evidence that shows the role of the dlPFC (sometimes with specific lateralization to the right hemisphere) related to the compliance of social norms, self-inhibition, and cognitive control (75). Following the preceding interpretation of frontal activity, theta activity in the EC might reflect a general response to adapt the behavior to a norm, in this case, related to suppressing inequity-aversion responses when inequity was advantageous to the other player. Evidence shows that social norms are reinforced by a third-party observer (75). Previous evidence using social observation has shown effects on how the brain encodes fairness, particularly in the context of social anxiety relative to the observation (75). Notably, we found behavioral and brain effects only in the empathy group and not in the Observer group. This suggests that the observer-experimental manipulation has to be conducted more strongly to produce more general brain and behavioral changes in the decision process. It can be suggested that future experimental designs include the measurement of personality traits to evaluate whether there are different sensibilities related to the influence of making decisions under observation since we found a correlation between behavior and theta activity within the observer group.

We have discussed two possible interpretations of our brain and behavioral results, one related to the effect of empathy-related responses associated with a person in need and another related to a more general compliance-to-the-norm response. These two interpretations can be considered complementary. Recently, fairness-related normative decision-making in social interaction has been analyzed from the perspective of two complementary processes: an intuitive component and a deliberative one (76). The intuitive component is related to the responses to conflicts between self-interests and a given social context; it is usually related to emotional factors and is reported as the responsible component for altruistic punishment and general prosocial responses (73, 77–82). The deliberative component has been related to a reappraisal process of conflict evaluation between, for instance, unfairness-evoked aversive responses (norm enforcement) and self-interest concerns (76). The brain areas related to this component are the MCC, dlPFC, vlPFC, and dmPFC and are thought to contribute to developing flexible strategies within decision-making settings. Although the source analyses have to be interpreted with caution, we can at least point out that the brain activity associated with the EC is in line with the idea of an early component associated with the inclusion of the “other” via the emotionally related component and that this response may have been reinforced by a norm-compliance adjustment.

Our investigation is subject to some limitations that need to be considered. Firstly, although the decision-making process in both experimental conditions took place in comparable settings, the physical presence of the observers may have caused some uncontrolled processing that could have introduced differences between the two conditions (e.g., perceived attractiveness, trustworthiness). Secondly, it has been shown that the power of frontal theta activity can be affected by factors such as anxiety (83), which were not directly controlled in our experimental design. Thirdly, for source estimation, it is important to note that only 64 electrodes may limit the strength of the claim regarding the cortical location of the oscillatory activity. Finally, further research is necessary using complementary measures that could help to shed light on different specific issues: the possible contribution of norm internalization and basal empathy tendencies to better disentangle the cognitive and emotional processing underlying prosocial decisions, the possible gender differences regarding empathy and norm-compliance tendencies.

Taken together, our results show that the inclusion of a person in need into the decision setting enhances prosocial behavior given by the inhibition of other-advantageous inequity aversion strategies. The modulation of theta brain activity indicated that these effects could be due to the enhancement of either empathy concerns or norm compliance. Due to the correlative nature of our experiment, it is necessary to inquire about the causal role of these possible brain and cognitive mechanisms by testing other experimental approaches, such as noninvasive brain stimulation.

## Data availability statement

The original contributions presented in the study are publicly available. This data can be found here: https://osf.io/YAHXJ/.

## Ethics statement

The studies involving human participants were reviewed and approved by the Ethical Committee of the Pontificia Universidad Católica de Chile. The patients/participants provided their written informed consent to participate in this study.

## Author contributions

CL, VL, and PB designed the experiment. CL and PB programmed the experiment, conducted the experiments, and analyzed the data. CL, PS-I, VL, and PB interpreted and discussed the results and wrote the manuscript. All authors contributed to the article and approved the submitted version.

## Funding

This work was supported by the Agencia Nacional de Investigación y Desarrollo de Chile (ANID) (PCHA/National Doctoral Program/grant 2016-21160667, FONDECYT 1211227; FONDECYT 11230607, FONDECYT 1211323; CONICYT PAI PAI77190047; and EQM150076).

## Conflict of interest

The authors declare that the research was conducted in the absence of any commercial or financial relationships that could be construed as a potential conflict of interest.

## Publisher’s note

All claims expressed in this article are solely those of the authors and do not necessarily represent those of their affiliated organizations, or those of the publisher, the editors and the reviewers. Any product that may be evaluated in this article, or claim that may be made by its manufacturer, is not guaranteed or endorsed by the publisher.
